# Limitations of democratic rights during the Covid-19 pandemic—exploring the citizens’ perception and discussions on dangers to democracy in Germany

**DOI:** 10.1007/s12286-023-00556-w

**Published:** 2023-02-14

**Authors:** Alexia Katsanidou, Marianne Kneuer, Felix Bensmann, Dimitar Dimitrov, Stefan Dietze

**Affiliations:** 1 GESIS—Leibniz Institute for the Social Sciences, Unter Sachsenhausen 6–8, 50667 Cologne, Germany; 2 Institute for Sociology and Social Psychology, University of Cologne, Cologne, Germany; 3 Institute of Political Science, Technical University Dresden, Dresden, Germany; 4 Heinrich Heine University Düsseldorf, Düsseldorf, Germany

**Keywords:** COVID-19, Democracy, Democratic rights, Twitter, Panel Survey

## Abstract

The governments’ mitigation measures to fight the COVID-19 pandemic are unprecedented in our post-war history. For overcoming this crisis, citizens were expected to act in compliance with these measures in order to control the spread of the virus and keep public health systems functional. This call for protecting the public health at the same time confronted citizens with several and severe limitations of their democratic freedoms and rights: confinement, restriction on freedoms of movement, religion, specific provisions for public protest and finally also limitations to the right of education by school closures. This paper analyzes how citizens perceive the threat the COVID-19 pandemic and especially the mitigation measures posed for democracy. We assume that pandemic waves and pandemic fatigue have an impact on the perception of threat. To see the overall societal picture, we exploit a large-scale archive of online discourse on Twitter out of which we extract democracy-related discourse with the same temporal and geospatial coverage for our investigation. From that data source, we apply computational methods to extract time series data reflecting aggregated opinions and their evolution over time concerned with the correlation of attitudes towards democracy. We them move deeper using a longitudinal panel survey we conducted in November/December 2020, March/April 2021, and July/August 2021. to have a view of the relationship between citizens’ socio-economic status and basic political attitudes. Our multi-method analysis bases on the German case and covers the period from December 2020 to August 2021.

## Introduction

The governments’ mitigation measures to fight the COVID-19 pandemic during the years 2020 and 2021 have been unprecedented in the post-war history. Public life came to a standstill, the likes of which had never been seen on this global scale since World War II and possibly even before. The measures, which very quickly diffused as best practices, included curfews and school and workplace closures, cancellation of public events, restrictions on public gatherings including religious celebrations, sports activities, closures of public institutions, stores and businesses, restrictions on movement, and international travel. All these measures implied the limitation or even suspension of political rights and civil liberties including freedom of assembly, the exercise of religious rights, freedom of movement, and not least the right on education (Hale et al. 2020).

These extraordinarily rigid reactions by governments—often possible only after declaring the state of emergency—raised the concern that these measures that without any doubt seriously limited individual freedoms would undermine democracy (Edgell et al. 2021; Lewkowicz et al. 2022). Two groups of political leaders are especially likely to exploit the pandemic for their purposes (Kneuer 2022): Firstly, political leaders with an illiberal approach who had engaged in democratic erosion already before the pandemic, dismantling democratic institutions, principles and practices, instrumentalized the pandemic to neutralizing the legislative and expanding executive powers (e.g. Victor Orbán, Jair Bolsonaro, Narendra Modi, the PiS government in Poland). Second, the reins were tightened further in countries under authoritarian rule before the pandemic, including cases such as China, Russia, Belarus, Venezuela, Philippines, Egypt, and Eritrea.

Understandablly the overwhelming part of the literature concentrated on the executive and its responses. What has hardly been studied so far, however, is the extent to which interference with democratic rights influences attitudes toward democracy (Alsan et al. 2021). This paper therefore fills a gap and examines if and how citizens perceive the threat for democracy during the Covid-19 pandemic and how they discuss this potential threat. This study is guided by four research questions:

1. Did citizens perceive a threat to democracy during the pandemic?
2. Are citizens satisfied with the way democracy worked in Germany during the pandemic?
3. Did this perception change over time, and if yes, in which direction?
4. And how is this citizens’ perceptions about democracy related to their socio-economic status, their political attitudes and the trust in their government?

Studies on the early phase of the pandemic point out a relatively broad support for the measures including those which limited democratic rights; “it seems that citizens understood that lockdowns were necessary and rewarded the responsible for them” (Bol et al. 2021, p. 498). We assume, however, that as the time went by pandemic fatigue had a negative influence on wide acceptance. Indeed, there is a first evidence for “elasticity” in the trade-off between health insecurity and civil liberties (Alsan et al. 2021, p. 25). Therefore, our assumption is that the willingness to comply with the democratic limitations change over time.

Our multi-method analysis of the German case covers the period from December 2020 to August 2021. We rely on two data sources: first, in order to give an overarching answer to the first three research question we explore public debates on Twitter. For this, we exploit a large-scale archive of online discourse on Twitter, consisting of approximately ten billion tweets captured through a continuous archiving effort since 2013 (Fafalios et al. 2018) out of which we extract a total of 56K tweets of relevance for our investigation. From that data source, we apply computational methods to extract time series data reflecting aggregated opinions and their evolution over time concerned with the correlation of attitudes towards democracy and health risks throughout the progression of the COVID-19 pandemic. Giving also some hints about temporal development but mainly in oder to capture the behavioural dimension as asked in our fourth research question we use a longitudinal panel survey conducted in November/December 2020, March/April 2021, and July/August 2021 (Katsanidou et al. 2021). On these data we run a descriptive analysis.

This paper provides a descriptive account on the development of perceptions and public debates of German citizens during the first and second Covid-19 pandemic wave in regard to possible damages to democracy. Our results show that the pandemic concerns for democracy were higher around May 2021, in comparison to December 2020, but they calmed again in August 2021. We also find significant differences among age and educational groups, and clear indications of the politicization of the pandemic, where supporters of AfD, FDP and Die Linke show more dissatisfaction with democracy than the supporters of the rest of political parties. A further important takeaway from the Twitter analysis (measured through tweet frequency and polarization of tweet sentiments) is the increased concern about democracy and that concerns are getting more emotional throughout the course of the pandemic. Thus, as the pandemic took its course, the public discourse on Twitter became more polarized.

Our study makes an important contribution to the research debate on the implications of the Covid-19 responses and how the citizens’ perceived the tension between the measures to protect health and the possible damages to democracy. The relevance of examining this trade-off lies in the fact that other crisis may also imply limitations to citizen rights. For political science research as well as for policy makers it remains of upmost importance to understand how far citizens are willing to accept democratic limitations without losing their belief in democracy.

## Democratic health in the Covid 19 pandemic—taking stock

Τimes of crisis are also times of executive power. A broad part of the early literature on COVID-19 pandemic therefore concentrated on the executive branch, in what way and with what measures governments responded to the outbreak and the course of the pandemic and how efficient these measures were. Part of the attention to the executive branch was directed at the question of what consequences declared states of emergency, as well as crisis management measures, would have on the health of democracy. The Varieties of Democracy research group generated two indices—the Pandemic Backsliding and Pandemic Violations of Democractic Standards Index—(Edgell et al. 2021) that measures changes in democracy quality and captures to what extent democratic standrads are violated by government responses. On top, the extent to which actions taken in the context of pandemic response were appropriate and proportionate, and to that extent proceeded within a “democratically sound” framework, was also observed with respect to established democracies (Edgell et al. 2021; Lewkowicz et al. 2022). A further, but smaller thread of literature addressed variation in government responses within the group of democratic countries (Engler et al. 2021). All in all, the literature on Covid-19 pandemic as a whole, but also in relation to the aspects of democratic constraints, very much targeted the macro perspective and the executive dimension.

Regarding the micro level perspective, what has been examined is compliance with government mitigating measures and also the the effect of trust on compliance. Given the knowledge about the positive relationship between political trust and health policy compliance during epidemics (Siegrist and Zingg 2014; Blair et al. 2017), several studies have been produced on the basis of the assumption that trust also played a role for the acceptance of the anit-Covid measures, especially as they were far more restraining than in other pandemic situations (e.g. Han et al. 2021; Gozgor 2020). Gonzor specifically looks at the sociodemocraphic breakdown for trust and identifies people with higher age and healthy people as those who trusted governments while those with higher education do less (Gozgor 2020, p. 573). These findings have to be taken with some caution, however, as the analisis only covers the lockdown period. For Germany, Jäckle et al. (2022) confirm the effect of political trust on mitigation measures. People who trust political leaders and institutions are clearly more likely to accept the arguments of the government when it comes to interventions aiming to tackle the pandemic, even if these represent encroachments on personal liberties (Jäckle et al. 2022, p. 19). Moreover, the authors detect that this positive effect of trust on the acceptance prevails in liberal minded persons and clearly decreases for authoritarian people. Another perspective looks behind the motives of the high levels of trust during the first and acute wave of the pandemic. Thus, Schraff argues that as the pandemic enters the phase of exponential growth in Covid-19 cases, “citizens start to rally around their political institutions as a lifebuoy” (Schraff 2020, p. 1007) due to the high level of uncertainty in the intense phase of the first wave. Schraff does observe an increased diffuse support during this time period, but holds that it was driven by the intensity of the crisis and not the specific government measures (Schraff 2020, p. 2015).

Most of the work on trust only covers 2020 or even only the lockdown period (Gonzor 2020) which might distort the results as such a extreme situation as a curfew is might rather increase trust while loosening measure then might decrease it again. This assumption is corroborated by the Cosmo Project (COVID-19 Snapshot Monitoring), a highly relevant source on public trust in Germany which monitored whether and how trust in institutions changes over the pandemic, but was especially interested in the two health institutions that were giving evidence based information—the Robert-Koch-Institute (RKI) and the Bundeszentrale für gesundheitliche Aufklärung (BZgA) (Eitze et al. 2021). The Cosmo data show that trust in RKI and BZgA was especially high during the curfew (March to May) and then decreased.

Finally, fewer studies dedicated themselves to the possible threat to democracy. There is some work done on this matter on the first wave of the pandemic (Alsan et al. 2021; Arceneaux et al. 2020). In all, regarding trust as well as effects on democracy in a broader way, there have been few studies to date that capture a longer period of inquiry in terms of citizens’ perceptions of democratic health. This longer view, however, is significant because both the length of the restrictions and the fact that they have been repeated may naturally heighten concerns about damage to democracy. Other actions taken by the German federal government over the course of the 2020 and 2021 pandemic years may also have contributed to heightened or even dissipated doubts about primarily long-term damage.

## The “democratic imposition”—the pandemic and democratic constraints in Germany

Interestingly, in Germany, Chancellor Merkel herself pointed out the problem with the restrictions adopted in connection with the pandemic, calling them a “democratic imposition” (Merkel 2020a, b) on several occasions. She used this phrase for the first time in her government statement in the Bundestag in April 2020, i.e., relatively at the beginning of the pandemic, emphasizing that the restrictions, including on personal liberties, were only acceptable if the reasons remained comprehensible and objections were permitted (Merkel 2020a). In fact, there had never been such a scale of restrictions on fundamental rights in Germany before.

**Fig. 1:**
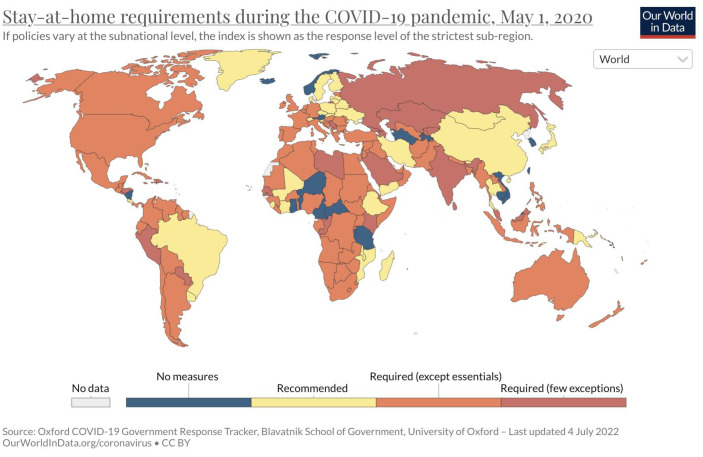
Stay-home requirements worldwide as of May 1 2020

**Fig. 2:**
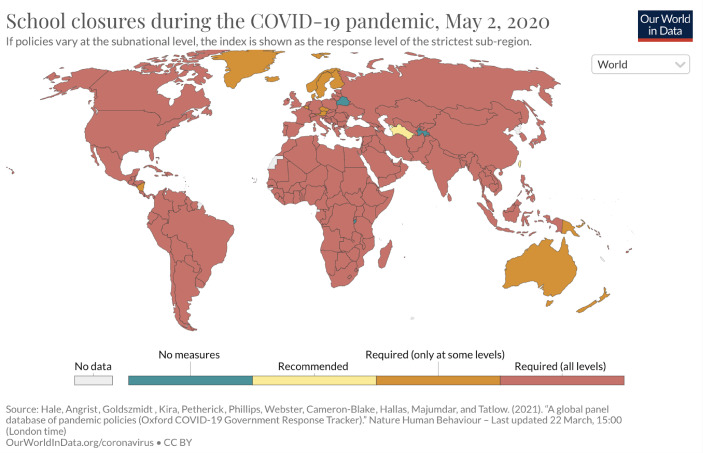
School closures worldwide as of May 2, 2020

Even if there was a variation in the governments’ responses, certain instruments did diffuse as best practices, especially curfews (Fig. 1) and school closures (Fig. 2) as well as workplace closures, cancellation of public events, closing of workplaces and public restrictions on public gatherings including religious celebrations, sports activities, closures of public institutions, stores and businesses, restrictions on internal movements, transport as well as international travel controls or even travel bans. All these measures implied the limitation or even suspension of political rights and civil liberties including the freedom of assembly, the exercise of religious rights, freedom of movement, and not least the right on education. Although Germany took harsh and limitating measures, the Pandemic Violations of Democratic Standards Index states, that Germany did not belong to those states that displayed such violations. Actually, Germany is ranked within the group of thirteen countries with the lowest scores for such violations worldwide (Edgell et al. 2021, p. 5).

## Satisfaction with democracy, trust, socioeconomic and political position

In this paper we follow two distinct paths: on one hand side and more generally, we look at the satisfaction with democracy; on the other hand side and more specifically, we examine the perception of threat to democracy. Even against the background of the account that the German government did not violate democratic standards, German citizens can have a different view on the performance of the government in terms of democratic restrictions during the pandemic. How did they perceive the state of democracy and possible changes and how satisfied were they with their democracy during the pandemic? Concerning satisfaction with democracy, we rely on the commonly used understanding of favorable attitudes and predisposition stimulated by outputs that are perceived by members to meet their demands as they arise or in anticipation (Easton 1965, p. 273). This specific support is closely related to the authorities of the political system and their output (Easton 1975, p. 437f.). The citizens evaluation of the authorities decisions, actions, policies and utterance form the basis for the specific support for democracy.

In times of crises, citizens are much more aware of their authorities and direct clear expectations to their governments and their problem solving capacities. Citizens especially expect to be kept safe and they demand actions and viable responses from their government in order to present viable responses to the crisis. Moreover, recently people “have become at once more fearful and less tolerant of major threats to public health, safety, and prosperity” (Boin et al. 2017, p. 14). Thus, the output sensitivity in times of crisis is relatively higher. Satisfaction with democracy thus is supposed to reflect the overall assessment of the government’s crisis response. In contrast, the perception of a possible damage to democracy refers to those measures that actually are related to limitations of democratic rights. It is important to mention that one cannot be sure wether satisfaction with democracy is influenced only by the crisis (in this case the pandemic). There might be other factors at play. However, the salience of the crises allows us to draw some conclusions even if there is not a full certainty that there is a causal relationship at play.

Confronted with a crisis, citizens will attribute high salience to solutions that are presented as soon as possible. Thus, in a first moment or phase of the crisis, this immidiate response of a government is required objectively but also subjectly by the public. As already mentioned, the acceptance of citizens in several countries of the crisis response in the acute months of the pandemic was quite high, also including the acceptance of harsh restrictions such as curfews, school closing etc. But what can appear pertinent in a certain situation can be evaluated differently in another. Thus, the longer democratic rights have been restricted, the greater the possibility that citizens might worry about long-term damage to their democracy. This perception however also can be influenced by the intensity of the threat. In this case, higher exposure to health risks lead to greater willingness to sacrifice rights and freedoms (Alsan et al. 2021, p. 25).

The distribution of the population in terms of their levels of satisfaction with democracy fluctuates based on various factors displaying that not all citizens are satisfied at the same time. Literature on satisfaction with democracy has shown that citizens with higher socio-economic status show higher levels of satisfaction with democracy, as they have higher cognitive skills and resources. This can be applied also in the pandemic, as there was a clear division among people who had the resources to be more careful but at the same time exercise some democratic rights, and also the cognitive skills to understand the scientific and societal need to accept mitigation measures.

The evaluation of democratic performance and the level of satisfaction with democracy is largely based in some sort of rational calculation (Downs 1957) based on limited information (Popkin 1991) using cognitive shortcuts. An example is that judgements about government policy delivery are positively linked to satisfaction with democracy (Clarke et al. 1993). In sum, citizens are satisfied with democracy if they feel that the government is effectively dealing with the most important problem facing the country and if they think the government represents effectively their views and interests. Inevitably, these elements connect satisfaction with democracy with political trust.

Political trust is strongly related to perceptions of performance (e.g. van der Meer and Hakhverdian 2017) but it can also be rooted in perceptions about the democratic system and its performance (Norris 2011). It is clear that democratic performance based on high institutional quality, rule of law, respect for human rights, electoral integrity citizens and so on coincides with citizens demonstrating higher levels of political trust (Dahlberg and Holmberg 2014). Political trust was also associated with higher levels of compliance with the pandemic mitigation measures. There is a strong relationship between trust in political institutions and willingness to practice social distancing (in Italy Barari et al. 2020; in France Lalot et al. 2020; in Denmark Olsen and Hjorth 2020). This effect has been described as ‘rally around the flag’ dynamic (Hetherington and Nelson 2003). For those people who trust the government and also accept covid mitigation measures we can assume that satisfaction with democracy has higher levels than those who distrust government.

Satisfaction with the way democracy works is lower among anti-establishment and populist party voters than other voters (Rooduijn 2018) and it is long known that radical right wing parties mobilise on this lack of democratic satisfaction (Hooghe and Dassonneville 2018). In the setting of the pandemic there is good indication that political parties that mobilized against the mitigation measures are also the ones whose supporters would demonstrate lower levels of satisfaction with democracy. For example in the USA (Barrios and Hochberg 2020) and Brazil (Ajzenman et al. 2020) pro-government voters demonstrated less compliance with measures due to the skepticism that the head of government in the respective country Donald Trump and Jair Bolsonaro expressed at the severity of COVID-19 respectively. At the time of the pandemic in Germany the two government parties CDU/CSU and SPD were very supportive of the mitigation measures and so was Green Party. The more populist counterparts, AfD and Die Linke, as well as the liberal FDP as a representative of democratic freedoms were pausing more questions if not rejecting the whole pandemic mitigation set of measures. Specifically, for FDP supporters it became clear over the pandemic that they did not feel comfortable with state interference (Jäckle et al. 2022). It is important to note however, that Die Linke’s perspective was on its basis anti-elite. On the one hand the party supported the lockdowns and other mitigatin measures but they put the condition that this had to be done within the idea of solidarity and supported by welfare benefits for those who suffer (Die Linke 2020a, b). Solidarity was indeed a dominant frame used in the pandemic (Katsanidou et al. 2022). Thus, we expect that supporters of these three parties would be the ones with more problematic evaluations of democracy but not all to the same extent.

We can thus summarize our expections as follows:

### H1:

Satisfaction with democracy drops over time as the pandemic continues while perceived threat to democracy increases.

### H2:

Satisfaction with democracy is higher among higher educated citizens and perceived threat to democracy higher among less educated.

### H3:

Satisfaction with democracy is higher among citizens with high political trust and perceived threat to democracy higher among citizens with low political trust.

### H4:

Satisfaction with democracy is lower and perceived threat to democracy higher among supporters of AfD, FDP and Die Linke.

In what follows we give an overview of the debate on the public sphere on the issue of satisfaction with democracy during the pandemic as presented in Twitter, and then we move to a presentation of a longitudinal survey data collection in the same period.

## The Twitter data and analysis

### Data & methods

Οnline discourse enables the observation of interaction of with each other in real-life discourse environments. Data from social media discourse has been widely used recently to describe opinions and attitudes on a variety of topics, for instance, political views (Pasek et al. 2020), attitudes towards economic indicators (Conrad et al. 2021) and to complement traditional survey data (Stier et al. 2020). Other works derive consumer satisfaction from Twitter data (Daas et al. 2015) or use social media data to identify social perceptions of unrest and insecurity that can be compared with survey data (Salvatore et al. 2020).

Pasek et al. (2020) find that, while the long-term trends in these data were largely similar, there are substantial differences in short-term changes. Further, mining of online discourse uncovers self-expressed sentiment and opinions as part of informal online discourse rather than explicitly stated attitudes in formal survey settings. We use them thus as complementary to survey measures as automatically mined sentiment from online discourse presents an added value as in combination with survey data. Together they provide a comprehensive understanding of solidarity attitudes and their evolution over time.

In order to complement survey data and enable the investigation of distinct discourse dimensions and their interdependencies, we exploit a long-term Twitter archive underlying TweetsKB^1^ (Fafalios et al. 2018), based on continuously capturing a random 1% sample of the Twitter stream using the Twitter streaming API. The crawler has been established in 2013, having continuously crawled more than 11 billion tweets until December 2021. The analysis of Twitter discourse poses challenges due to its specific characteristics. These include its heterogeneity with respect to reflected user demographics, content and language, its scale and its strong dependence on societal events, where particular posts tend to reflect not stable long-term attitudes but dynamic expressions of opinions reflective of specific events and time points. Hence, in order to investigate the evolution of discourse and opinions as required for our analysis, we have to process data by (a) generating seed lists representative of the variables under investigation and retrieving Twitter data for each variables, matching spatial and temporal regions under investigation, (b) computation of sentiments in order to automatically classify individual posts along their emotional connotation and (c) aggregating time series which reflect the evolution of opinions and attitudes over time.

The complete Twitter archive underlying TweetsKB, denoted as *TweetsKB*_*Arch*_, contains the entire archive of the captured Twitter stream including the actual text body of all tweets, on which the processing of this work has been conducted. Hence, in order to investigate the hypotheses, we first identify suitable subsets of *TweetsKB*_*Arch*_, using semi-automatically generated seed lists that represent democracy concerns in connection with the COVID-19 discourse. Seed lists are designed to ensure (a) a match between seed terms and the vocabulary used in Twitter discourse and (b) high relevance of seed terms for the respective issue of concern. For the time between 20.02.20 and 20.02.21, we select all German language tweets from *TweetsKB*_*Arch*_ (see the detailed procedure in the Appendix A).

Similar to the methodology applied for creating TweetsKB, we calculate the sentiment scores for all of the retrieved tweets using SentiStrength (Thelwall et al. 2010), a dictionary-based sentiment analysis algorithm that is shown to generalize and scale well across large data volumes. As we focus on the German Twitter COVID-19 discourse we calculated the sentiments scores using German dictionary. For each tweet the algorithm outputs a positive and a negative sentiment score ranging from −1 (not negative) to −5 (extremely negative) for the negative and from 1 (not positive) to 5 (extremely positive) for the positive sentiment part.

In order to investigate the trade-off between the issues of concern as required in your hypotheses we construct and analyze time series. We construct frequency and sentiment time series for each issue of concern by aggregating the number of tweets and the averaging the sentiment scores (positive and negative) for all tweets per day.

### Analysis

Following the approach described above and in more detail in Appendix A, we extract Twitter discourse based on different seed lists. Details of the obtained datasets are shown in Table 1.

**Table 1:** Descriptive statistics for all Twitter subsets reflecting democracy discourse in Germany using the approach described Appendix A

	SL 1	SL1 + COV	SL2	SL2 + COV	SL3	SL3 + COV	SL4	SL4 + COV
#Tweets with COVID term:	n.a.	332,096	n.a.	332,096	n.a.	332,096	n.a.	332,096
#Tweets with seed term:	203,033	13,390	141,181	8867	173,488	12,031	330,761	26,385
#Tweets (daily mean):	333.388	21.987	231.824	14.56	284.874	19.755	543.122	43.325
Kendall’s tau tweet frequency:	0.235	−0.033	0.172	−0.004	0.194	−0.001	0.381	0.1
Mean positive sentiment:	1.521	1.533	1.463	1.431	1.46	1.449	1.494	1.46
Mean negative sentiment:	−1.602	−1.68	−1.622	−1.671	−1.618	−1.658	−1.609	−1.677
Mean consolidated sentiment:	−0.041	−0.077	−0.079	−0.132	−0.079	−0.109	−0.057	−0.114
Kendall’s tau positive sentiment:	0.12	0.159	0.067	0.117	0.065	0.083	0.058	0.144
Kendall’s tau negative sentiment:	−0.022	−0.136	−0.01	−0.141	−0.003	−0.155	0.034	−0.11
Kendall’s tau consolidated sentiment:	0.096	−0.028	−0.026	−0.076	0.031	−0.095	0.059	−0.032

In Table 1, SLX refers to seed list X (Appendix), e.g. seed list 1, where SLX + COV refers to an application of seed list X with the additional constraint, that the resulting tweets contain at least one of the COVID-19 terms: *corona, covid, sars-cov‑2, sarscov2, sars_cov_2*. Overall, we observe that tweet frequency drops through the second constraint of requiring COVID-19 terms, i.e. the total amount of tweets about solidarity discourse with explicit references to COVID-19 terms decreases over time. With respect to tweet frequency trends, we will ignore the SLX + COV seed lists on the basis that the overall explicit mention of COVID19-terms on Twitter has been decreasing steadily throughout the pandemic. However, that is not to be taken as indicator of decreasing relevance of COVID-19 but is an artefact of the discourse data, where increasingly obvious contextual information, i.e. some relation with the pandemic context, is not mentioned explicitly.

**Fig. 3:**
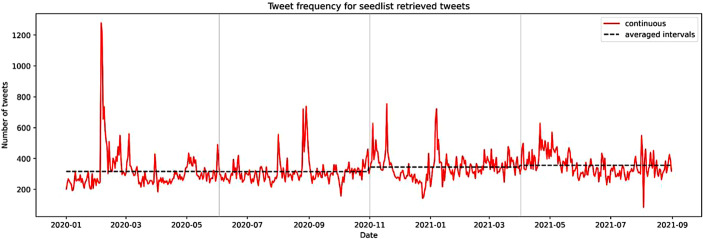
Evolution of democracy discourse in Germany (SL1) over time

As shown in Fig. 3, there is a strong fluctuation in the data driven by a range of societal events and policies. Focusing on the dashed line that denotes the increasing mean frequency (316.861, 314.483, 344.927, 356.32) within equally sized time intervals, we observe an increasing trend in democracy discourse, indicating an increasing concern for democracy in the population. This observation is supported by all twitter data subsets under investigation. While a detailed discussion of peaks and fluctuations and their correlation with societal events is out of scope of the paper, it is worth noting manual exploration of tweets reveals that, for instance, the largest peak in February 2020 is driven by the first lockdowns in Germany.

**Fig. 4:**
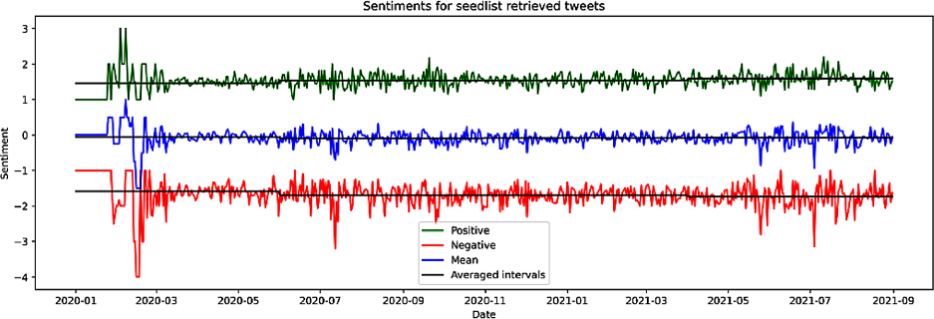
Evolution of sentiments of democracy discourse in Germany (SL1 + COV) over time

With respect to sentiments expressed in Twitter discourse, for more precise results, we refer to SL1 + COV and show the evolution over time in Fig. 4. Here, the green line represents the average positive sentiment, the red line the average negative sentiment and the blueline the mean of positive/negative sentiments. We observe strong fluctuations (upper plot) and an increasing polarization over time, where positive sentiments get more positive (Kendall’s Tau = 0.159) and negative sentiments get more negative (Kendall’s Tau = −0.136) with little (negative) effect to the overall mean (Kendall’s Tau = −0.028). For computation of trends, we split the time series into four equally long time intervals (with the last one storing one extra day because of the division) and applied trend detection to each interval. This way we get a measure of what the trends are and how they change over time, while the intervals keep a meaningful length of *n* = 152 (153). The trend detection was done computing each interval’s correlation with a continuously increasing time series representing the same resolution and length, i.e. (1, 2, 3, … *n*). As correlation coefficient we used the Kendall’s tau implementation from scipy 1.7.3.

Summarizing our findings with respect to the research questions, we indeed observe an increased concern about democracy during the pandemic, where concern—measured through tweet frequency and polarization of tweet sentiments—increases over time, indicating that concerns related to democracy are increasing and getting more emotional throughout the course of the pandemic. This is an indication in support of our first hypothesis that satisfaction with democracy drops over time as the pandemic continues while perceived threat to democracy increases.

## Discussion of multi-method approaches involving Twitter data

Online discourse enables to observe how attitudes interact with each other in real-life discourse environments. Twitter is not the only source of online discourse but is widely used for sharing opinions on societal and political matters and part of the news cycle where news from online media are picked up in Twitter discourse and vice versa. Thus, data from social media discourse has been widely used to mine opinions and attitudes on a variety of topics, for instance, political views (Pasek et al. 2020), attitudes towards economic indicators (Conrad et al. 2021) and to complement traditional survey data (Stier et al. 2020). Other works derive consumer satisfaction from Twitter data (Daas et al. 2015) or use social media data to identify social perceptions of unrest and insecurity that can be compared with survey data (Salvatore et al. 2020).

Whereas correlations between survey-based and web-mined opinions and attitudes are apparent and prior work has demonstrated that automatically mined emotions can be aggregated towards meaningful time series data representing the evolution of actual attitudes towards topics of interest (Conrad et al. 2021), both data sources differ in various ways (Joseph et al. 2021). Different to survey data, online discourse is able to capture short-term fluctuations in public opinion and their dependencies with societal events. Pasek et al. (2020) find similar long-term trends in these data but substantial differences in short-term changes. Further, mining of online discourse uncovers self-expressed sentiment and opinions as part of informal online discourse rather than explicitly stated attitudes in formal survey settings. Although opinion trends mined from online discourse necessarily provide an imperfect reflection of public opinion, automatically mined sentiment from online discourse complement survey-based data to jointly provide a comprehensive understanding of solidarity attitudes and their evolution over time. It is important to acknowledge that any step during the data gathering and processing pipelines introduce their own biases, including the techniques used for crawling, retrieving data, e.g. for specific variables, georeferencing or sentiment analysis. Considering these inherent biases, different data and processing methods can uncover additional and complementary perspectives, able to improve the understanding of attitudes towards specific social science variables over time.

## The survey data and analysis

### Data & method

We now move to the longitudinal survey data for the same time period. The data were collected during the SAFE-19 project, funded by the federal ministry of education and research (Katsanidou et al. 2021). It is an online, non-probability panel using the Respondi Access Panel^2^ based on population quotas of gender and age and it contains people living in Germany (German and foreigners) between 18 and 69 years. During the respondent recruitment, quotas were applied to achieve a sample that mirrored the composition of Germany’s resident population in terms of age, gender and education level (university entrance qualification vs. lower levels of education) distribution. Notwithstanding these efforts, it must be stressed that the project used a non-probability-based sample. Consequently, the reported distributions, despite being representative in terms of age, gender and education level, cannot be generalised to the overall population of Germany. A more detailed presentation of demographic variables can be found in Appendix B. Three waves were collected, the first in end of November to early December 2020, the second at the end of March 2021 and the third at the end of July 2021 reflecting three distinct phases of the pandemic. During the waves most of the questions remained the same to see differences in the progression of the pandemic. The initial sample of wave 1 consisted of 2250 individuals. The sample used in this paper contains 1166 participants for each wave in the balanced panel.

**Fig. 5:**
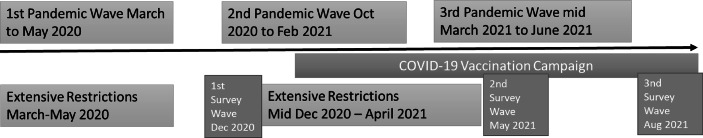
Timeline COVID-19 pandemic

To refresh the collective memory, the first wave of our data collection took place shortly before the second pandemic wave took hold, and before the extensive restrictions were imposed again. The German population experienced a longer period of things being almost back to normal and anticipated further normalizations based on news about the upcoming vaccination campaign. The second survey wave took place in the midst of the third pandemic wave, when the extensive restrictions were just waved, but significant restrictions were still in place. The third wave of our survey took place in a time of relative calmness, where the third wave had passed and restrictions were minimal. Important is also that the second and third wave of our panel study took place as the vaccination campaign was unfolding. As of May 1st 2021 only 7.93% of the German population was vaccinated while the proportion of the population vaccinated jumped to a 52.28% by August 1st 2021 (Fig. 5).^3^

The main question of this paper is how citizens perceive the threat for democracy during the Covid-19 pandemic. To assess this, we look into two variables, the level of satisfaction with democracy, and the perceived damage done to democracy. Both variables are not looked in isolation but keeping in mind the time perspective as well as other individual level characteristics such as age, level of education, gender, party preferences and trust in government.

### Analysis

In a first step our anlysis addresses satisfaction with democracy and the perceived damage to democracy, offering a first overview of the time perspective. Satisfaction with democracy is asked in a scale from 1–7, where 1 stands for “very unsatisfied” and 7 for “very satisfied”. It becomes clear that satisfaction with democracy drops in May 2021 when compared with December 2020, but it takes up again in August 2022 but it does not achieve the same levels as in December 2020. The perceived damage to democracy is coded in the opposite way, where 1 stands for “no damage” and 7 for “very severe damage”. Here, very much in line with the findings on satisfaction with democracy, we find that the perceived damage to democracy increases in May 2021 but drops again in August 2021 not quite reaching again the initial levels of December 2020 (Table 2). These findings show that H1 can only partially be confirmed, as in the duration of the pandemic satisfaction with democracy dropped significantly but did not fully recover, while the perceived damage to democracy increased only to decrease again, but not to the initial levels. This corroborates to some extent with the results from the twitter analysis, where it becomes clear that it is not the levels of satisfaction with democracy that drop so much, but rather the polarization between those who are satisfied with democracy and those who are not that deepens.

**Table 2:** Means per wave

	Wave 1 Dec 2020	Wave 2 May 2021	Wave 3 Aug 2021	Differences between waves W1 and W2, W2 and W3 Statistically Significant (t-tests)
Satisfaction with democracy	4.34	4.02	4.23	Yes
Damage to democracy	4.4	4.85	4.6	Yes

**Fig. 6:**
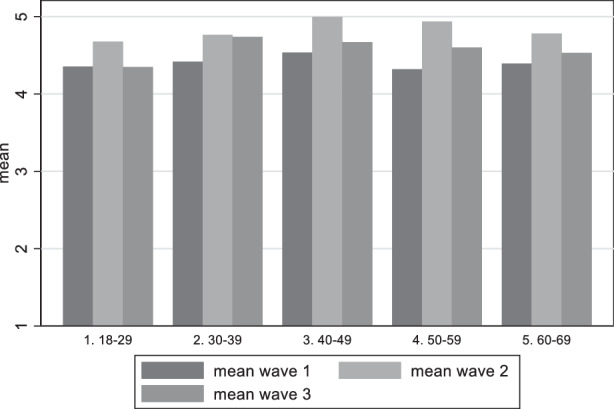
Variation of satisfaction with democracy among age groups

As our sample is non-probabilistic (representative but not random) this information has to be taken with a grain of salt. In the next step, looking at different demographic groups (age and gender) we find significant differences among them regarding satisfaction with democracy. Figure 6 shows the variation in the mean answers on the question on satisfaction with democracy among age groups. There are two main observations. Firstly, there seems to be a rather weak U‑shape effect of age. The youngest and the oldest population groups are the ones that are more satisfied, while the middle aged are the ones with the lower scores in satisfaction with democracy. Secondly, wave two shows striking differences to the other two waves. Knowing that in wave two satisfaction with democracy drops, using Table 1 we can locate this drop mainly in the age groups 40–49 and 50–59.

**Fig. 7:**
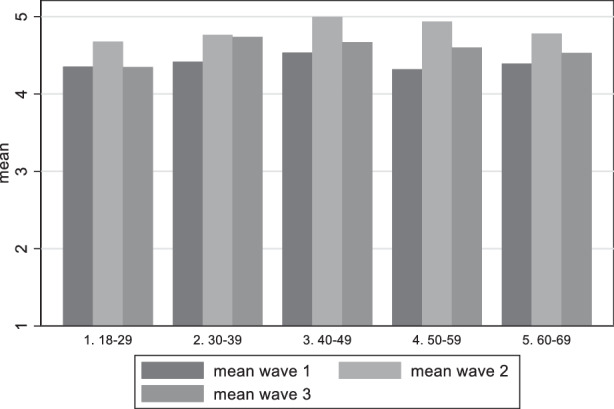
Variation of perceived damage to democracy among age groups

The same exercise is now applied to the perceived damage to democracy variable (Fig. 7). Breaking down the responses of those who do preceive a damage to democracy (5–7 in the scale), we find that the share clearly rises in the course of the pandemic. While in the first wave 46.6% of the repondents felt the threat for the functioning of democracy, this value increased to 57, 49% in the second wave and even to 64.75% in July/August 2021. Those with the highest perception that democracy has been damaged are in the age groups 40–49 and 50–59. Important to note is that in the first wave the group 50–59 has lower scores but experiences the highest increase in wave 2, showing that they were the group most affected in the second wave. These differences are small but statistically significant.

**Fig. 8:**
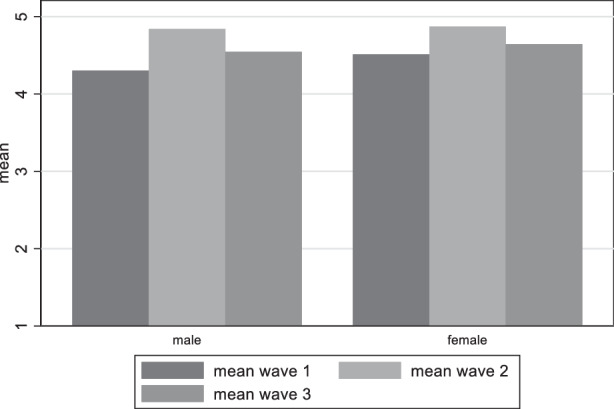
Variation of satisfaction with democracy among gender groups

**Fig. 9:**
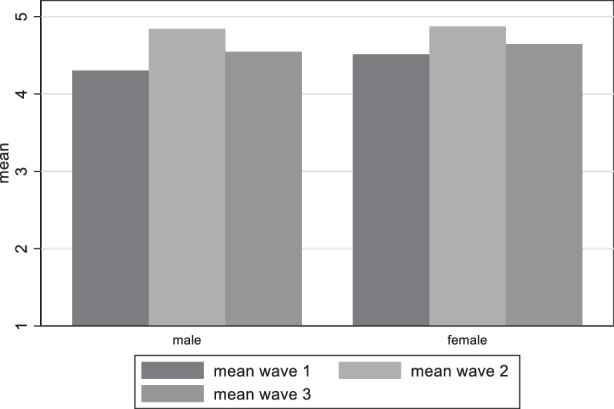
Variation of perceived damage to democracy among gender groups

Turning to the question if there is a difference in the distribution of satisfaction with democracy between genders, we see in Fig. 8 that the differences among genders are so small that are not statistically significant (t-tests conducted). Similarly, Gender does not seem to play a role for the perceived damage on democracy, as the distribution between genders is very similar and the differences statistically insignificant, apart from the first wave, where more women believe that democracy has been damaged, than men (Fig. 9).

**Fig. 10:**
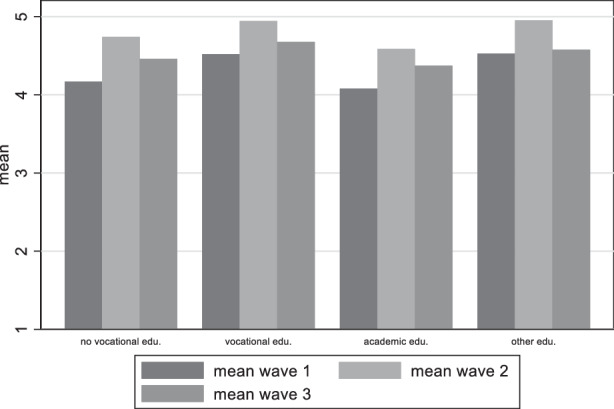
Variation of satisfaction with democracy among educational achievement groups

**Fig. 11:**
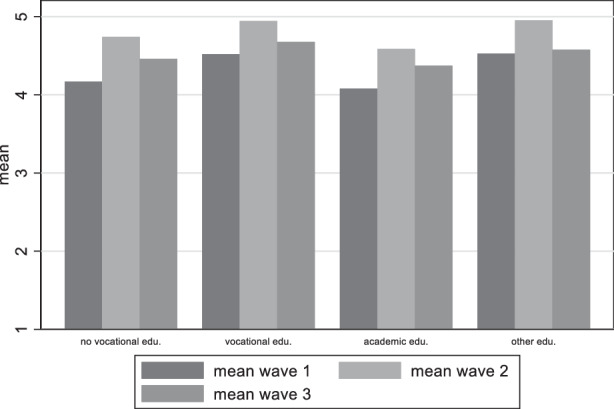
Variation of perceived damage to democracy among educational groups

Our hypotheses were based on the differences among educational groups, supporters of political parties and those who are not trusting of the government as opposed to those who were not trusting. Turning our attention to the division of the results on the basis of educational achievement to find indication to support or reject our second hypothesis, Fig. 10 shows that in general those with vocational education have lower satisfaction with democracy than those with academic education, and this finding remains constant across waves. Figure 11 gives an overview of the distribution among educational groups for the variable on the perceived damage on democracy. Here too, those with vocational education are the ones with the strongest belief that democracy has been damaged. We can thus accept our second hypothesis that claiming that Satisfaction with democracy is higher among higher educated citizens and perceived threat to democracy higher among less educated.

**Fig. 12:**
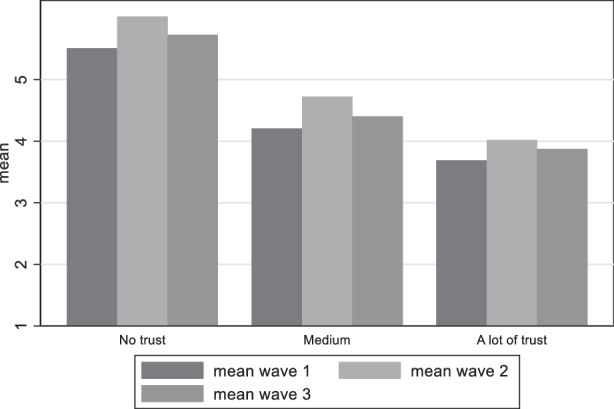
Variation of satisfaction with democracy among trust in government groups

**Fig. 13:**
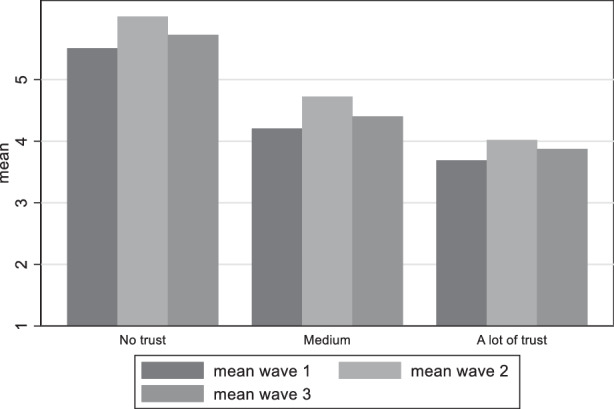
Variation of perceived damage to democracy among trust in government groups

Our third hypothesis distinguished between various levels of trust. Figure 12 demonstrates the differences in satisfaction with democracy among the groups with different levels of trust in government. It does not come as a surprise that among those with low trust in government satisfaction with democracy is notoriously low, while satisfaction is higher among those individuals claiming high trust in government. Political trust follows also the same pattern with the perceived damage to democracy as with satisfaction with democracy, where respondents with higher trust believe much less that democracy has been damaged (Fig. 13). We can thus accept our third hypothesis claiming that satisfaction with democracy is higher among citizens with high political trust and perceived threat to democracy higher among citizens with low political trust.

**Fig. 14:**
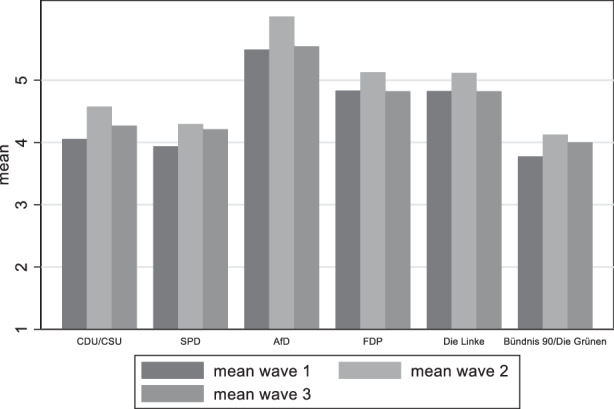
Variation of satisfaction with democracy among party groups

Looking now at the political variables, Fig. 14 presents the mean differences among political party groups. It becomes clear that the group with the lowest satisfaction with democracy includes individuals that would vote for the AfD. A second group with lower satisfaction includes the FDP and Die Linke voters. There are no differences among the CDU, SPD and the Greens in the first wave. In the second and third wave however, SPD and the Green supporters seem to be losing some of their satisfaction with democracy.

**Fig. 15:**
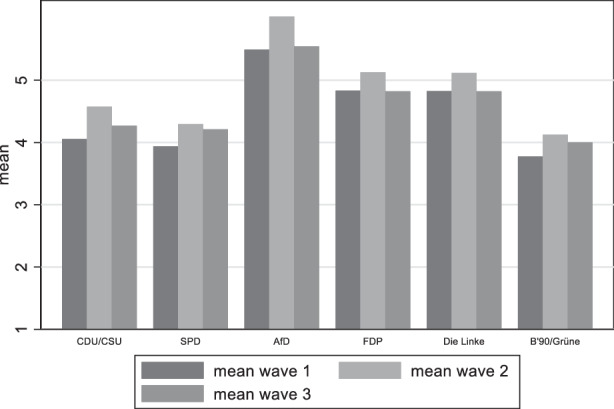
Variation of perceived damage to democracy among party groups

Moving on to the question to what extent there is a difference in the distribution of the belief that the pandemic will bring damage to the function of democracy among party groups, we see (Fig. 15) a similar picture as with satisfaction with democracy. Respondents with AfD preferences are those who believe more that democracy has been damaged, followed by FDP and die Linke supporters, thus confirming our fourth and final hypothesis.

## Discussion

Our first research question asking for the perception of German citizens regarding the threat for democracy can be answered in the following way: Indeed, Germans saw the threat for a damage to democracy and this perception increased in the course of the pandemic. The analysis showed that it is the less educated people and the age group between 40 and 60 which most perceive this damage. This result corresponds to the results on satisfaction how democracy worked during the pandemic. Interestingly, however, the voting preference is relevant insofar as respondents near to the AfD are the group which most are worried about a damage to democracy and this remains constant during the period of investigation. This result resonantes with the rare evidence of other countries, namely that citizens disadvantaged by income, education or race (in the USA) are less willing to sacrifice rights than their more advantaged peers (Alsan et al. 2021). Likewise, a group of “outraged” and “disappointed” seems to be solidifying, uniting certain characteristics such as concern about democratic freedoms, significant dissatisfaction with problem-solving ability during the pandemic, and a general dissatisfaction with democracy. This group also was shaped by more mistrust towards the government and the perception that the government is acting in an undemocratic way (Krause et al. 2020).

Our analysis additionally demonstrates that the preception of a damage to democracy increased the longer the pandemic endured, but also showed fluctuations based on the severity of the respective pandemic phase. We were able to identify in a more fine-grained way the concrete concerns of citzens regarding the damage to democracy, and here it is most interesting that it was not the fundamental political rights (religion, protest) where concern emerged or intensified, but more liberties on a personal level (social encounter, travels, and free movement). This suggests that citizens were quite able to endure restrictions on their basic democratic rights, but less able to endure constraints on their rights in everyday life.

The evidence of change over time is equally reflected in the Twitter data. Firstly, the Twitter discourse shows an increasing concern for democracy-related issues over time, expressed through increasing frequency of tweets within the considered pandemic time period. And secondly, the sentiments of the discourse also evolve over time: the positive sentiment getting more positive and the negative sentiments more negative. This can be interpreted as an additional sign of increased concern for democracy-related issues and is mirrored by trends observed through the other seed lists. But more than that it points to an increasingly polarized discourse regarding the concern for damage to democracy.

## Conclusion

This paper offers a descriptive account of the perceptions on democratic performance during pandemic years of 2020 and 2021 which were most relevant in terms of harsh restrictions. We used a dual methodological approach to achieve a birds-eye shot on attitudes on the micro level looking at the public discourse as it unfolded on Twitter before zooming to the individual level presentation of specific connections between socio-economic positioning, political attitudes, and perceptions of democratic performance. The findings relate to the period from the beginning of the pandemic until the summer 2021 and thus cover a broader time span than other studies.

As an important factor for the perception of democratic damage we detect time-relatedness (the longer the pandemic endured, the more concern on democratic damage increased), but also the intensity of the pandemic (increasing COVID-19 cases and deaths) as relevant factor for the fluctuation of satisfaction with democracy. In May 2021, respondents were less satisfied with the way democracy functioned in Germany in comparison to December 2020, but the trend did not hold. In August 2021 satisfaction with democracy went slightly up again. We also found that some elements of the socio-economic position matter. Those in the age categories 40–49 and 50–59, as well as those with vocational education (as opposed to academic education) show lower levels of satisfaction with democracy. Political trust has the expected positive relationship with democratic evaluation, while support for AfD, Die Linke, and FDP is connected to higher levels of democratic dissatisfaction and fear of democratic damage, and this relationship increased over time.

These findings, when put together, give an interesting picture of the German citizenry during the pandemic. An important takeaway is the fact that as the pandemic took its course on the one hand the public discourse on Twitter became more polarized. The positive sentiments over democracy became more positive and the negative ones more negative, which implies that as the time went by statements on democracy became more emotional. This finding can be supported also with the survey findings that show that, as the time went by, the democratic dimension of the pandemic became increasingly politicized and thus polarized.

The combined analysis from survey and twitter data show the way for future work. Twitter data offer an overview of the public debate that both reflects the mood of society, but can also shape perceptions of individuals. Results from survey analyses can corroborate the results of twitter analysis and offer a more into-depth analysis of global phenomena.

Our analysis contributes to the emerging topic of trade-offs in crises, especially those trade-offs that concern difficult choices regarding democratic rights and democratic standards. In view of the most recent crisis of climate and energy, again such trade-offs are looming. Therefore, more research on the citizens’ attitudes regarding their willingness to accept hardships and constraints is needed. Future studies should spread more light especially on the mechanisms of polarization and politicization of this topic.

## Appendix A

### Tweet retrieval through semi-automated seed lists

We exploit a long-term Twitter archive underlying TweetsKB^4^ (Fafalios et al. 2018), based on continuously capturing a random 1% sample of the Twitter stream using the Twitter streaming API. The crawler has been established in 2013, having continuously crawled more than 10 billion tweets until December 2020. TweetsKB itself represents a public endpoint of semantically annotated tweets, containing all relevant tweet metadata and extracted hash tags, URLs, and mentions lifted into an easy-to-use and interpret metadata schema, together with precomputed semantic annotations in the form of sentiment scores and named entity annotations precomputed using SentiStrength (Thelwall et al. 2010) and FEL (Blanco et al. 2015). In contrast, the complete Twitter archive underlying TweetsKB, denoted as *TweetsKB*_*Arch*_, contains the entire archive of the captured Twitter stream including the actual text body of all tweets, on which the processing of this work has been conducted.

In order to investigate the hypotheses, we first identify suitable subsets of *TweetsKB*_*Arch*_, we retrieve a subset that (a) addresses time period and geographic region of interest and (b) addresses democracy in connection with the COVID-19 discourse. Regarding (a) we obtain all German language tweets in the time period 20.02.2020–20.02.2021. Although existing geotagging approaches allow assigning a location to tweets, they exhibit relatively low accuracy while pre-trained on English tweets (Dimitrov et al. 2022). Using language as a proxy to country-related discourse instead of geotagging worked well as less than 7% of the manually examined tweets are German language tweets that do not address COVID-19 discourse in Germany, thereby providing a better precision that state-of-the-art georeferencing tools.

In order to address (b), we deploy semi-automatically generated seed lists that represent the democracy as an issue of concern. For that, we deployed different seed lists using different approaches. Seed List 1 was produced by through the initial source keywords *demokratie, grundrechte, freiheit*. Hereby, “*demokratie*” is the direct German translation of each issue of concern and a considered as the *primary *source keywords, whereas the remaining two source keywords in each issue of concern are *secondary *source keywords. We use case insensitive keywords to filter tweets. Subsequently, for a given source keyword, e.g., “*demokratie*” and its subset of tweets *S*, we apply part-of-speech (POS) tagging to the tweets’ texts and build a dictionary of all proper nouns, nouns, verbs, and adjectives. To determine semantic similarity of each term in the dictionary to the initial keyword, we use word embeddings (Bojanowski et al. 2017) pretrained on Common Crawl and Wikipedia^5^. Similarity is computed as cosine similarity of candidate terms to the vector representation of the source keyword (in this example “*demokratie*”). Experts manually inspect the resulting top-50 seed terms and create a final seed list by removing and replacing obviously irrelevant and unrelated terms from the automatically generated seed list for the *primary *source keyword with terms from the other two automatically generated list generated through the *secondary *source keywords. The final seed list contains 30 seed keywords used to filter tweets from *TweetsKB*_*Arch*_., a number which is high enough to ensure stable results for the subsequent analysis and reasonable topical coverage.

Manual examination from qualitative coders of a random sample of 100 tweets for each seed list showed that the semi-automatically generated seed lists were able to achieve on average an accuracy above 85% on the tweet retrieval task compared to domain expert seed list that achieved an accuracy around 60%.

Seed List 2–4 have been generated using a fully automated approach, where we introduce a weighting parameter α that determines the balance between term frequency and term similarity, where lower α indicates a stronger emphasis of term similarity. Seed List 4 in addition deploys stemming.

**Table A.1:** Semi-automatically generated seed list terms. (References: Dimitrov et al. 2022; Bojanowski et al. 2017)

Seed List 1	Seed List 2 (α = 0.2)	Seed List 3 (α = 0.5)	Seed List 4 (α = 0.2, stemming)
Demokratie	Demokratie	Demokratie	.*demokrat.*
Diktatur	Demokratische	Demokratisch	.*rechtsstaat.*
bürokratie	Demokratisch	Demokratische	.*parlamentar.*
Rechtsstaat	Rechtsstaatlichkeit	Demokrat	.*diktatur.*
Kapitalismus	Demokrat	Rechtsstaatlichkeit	.*föderalismus.*
Demokrat	Parlamentarische	Diktatur	.*zivilgesellschaft.*
Sozialstaat	föderalismus	Rechtsstaat	.*freiheit.*
Partizipation	Diktatur	Parlamentarische	.*totalitarismus.*
Marktwirtschaft	demokratieverständnis	Politische	.*autokrati.*
Faschismus	Parlamentarismus	Sozialismus	.*volksentscheid.*
Kommunismus	Rechtsstaat	Faschismus	.*sozialismus.*
Meinungsfreiheit	Antidemokraten	Liberale	.*polit.*
Demokratische	Autokratie	Pressefreiheit	.*pluralismus.*
Demokratisch	Staatsform	Meinungsfreiheit	.*liberal.*
Sozialismus	Zivilgesellschaft	Grundrechte	.*staatsform.*
Grundrechte	Freiheitliche	Kapitalismus	.*kapitalismus.*
Selbstbestimmung	Pluralismus	Menschenrechte	.*marktwirtschaft.*
Menschenrechte	Volksentscheid	Freiheitliche	.*versammlungsfrei.*
Nationalismus	Sozialismus	Altparteien	.*faschismus.*
Populismus	Antifaschismus	föderalismus	.*pressefrei.*
menschenwürde	Politische	Marktwirtschaft	.*altpartei.*
Zivilgesellschaft	Totalitarismus	Parlamentarisch	.*bürgerbeteil.*
Imperialismus	Undemokratisch	Zivilgesellschaft	.*nationalstaat.*
bürgerrechte	Liberale	Volksentscheid	.*wahlrecht.*
Volk	Kapitalismus	solidarität	.*rechtstaat.*
Pressefreiheit	Marktwirtschaft	Wahlrecht	.*sozialist.*
Menschenrecht	Versammlungsfreiheit	Antifaschismus	.*oligarchi.*
Gleichberechtigung	Pressefreiheit	Undemokratisch	.*volkspartei.*
Gewaltenteilung	bürgerbeteiligung	Freiheitlich	.*grundrecht.*
Freiheit	Faschismus	Antidemokraten	.*volksvertret.*

## Appendix B

﻿

**Table B.1:** Summary statistics of the survey data

Variable								
Gender	50.82% male	49.18% female	–	–	–	–	–	–
Age	21.07% 18–29 years	19.02% 30–39 years	18.67% 40–49 years	24.13% 50–59 years	17.11% 60–69 years	–	–	–
Place of residence	10.78% Baden-Wuerttemberg	14.42% Bayern	5.71% Berlin	3.89% Brandenburg	0.30% Bremen	2.76% Hamburg	8.07% Hessen	1.48% Mecklenburg-Vorpommern
Place of residence	9.10% Niedersachsen	21.6% NRW	5.46% Rheinland-Pfalz	1.03% Saarland	6.20% Sachsen	3.10% Sachsen-Anhalt	3.44% Schleswig-Holstein	2.66% Thüringen
Education	14.58% no vocational education	63.87% vocational education	17.33% academic education	4.22% other education	–	–	–	–
PID (Nov-Dez 2020)	32.10% CDU/CSU	13.15% SPD	13.93% AfD	7.35% FDP	11.18% Die Linke	22.30% Die Grünen	–	–

## Footnotes

1 STweetsKB is a large-scale corpus of semantically annotated tweets; see https://data.gesis.org/tweetskb/.

2 For further information on the Respondi Access Panel, see https://www.respondi.com/EN/access-panel.

3 Information taken from covidvax.live on July 5th 2022.
